# Spontaneous Tear of the Tibialis Anterior Tendon Presenting as a Pseudotumor

**DOI:** 10.5334/jbsr.3074

**Published:** 2023-04-27

**Authors:** Aseel Al-Musaedi, Filip M. Vanhoenacker

**Affiliations:** 1 AZ sint maarten ziekenhuis, BE; 2 AZ Sint-Maarten and University (Hospital), Antwerp/Ghent/Leuven, BE

**Keywords:** anterior tibialis tendon, tendon rupture, pseudotumor, MRI, ultrasound

## Abstract

**Teaching Point:** Spontaneous rupture of the anterior tibial tendon at the ankle joint may mimic a tumor.

## Case History

**Figure 1:**
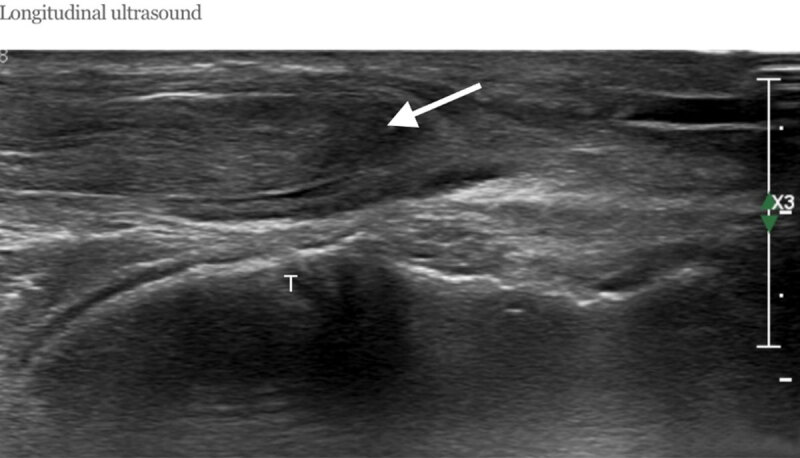


A 75-year-old man presented with spontaneous soft tissue swelling at the left ankle joint anteriorly. Longitudinal sonogram of the tibialis anterior tendon (TAT) of the left ankle joint showed a retracted, irregular, hypoechoic, and swollen proximal part of TAT and pre-existing tendinopathy. There was focal discontinuity of the TAT between superior and inferior retinaculum (Figure 1, white arrow T: tibia).

**Figure 2:**
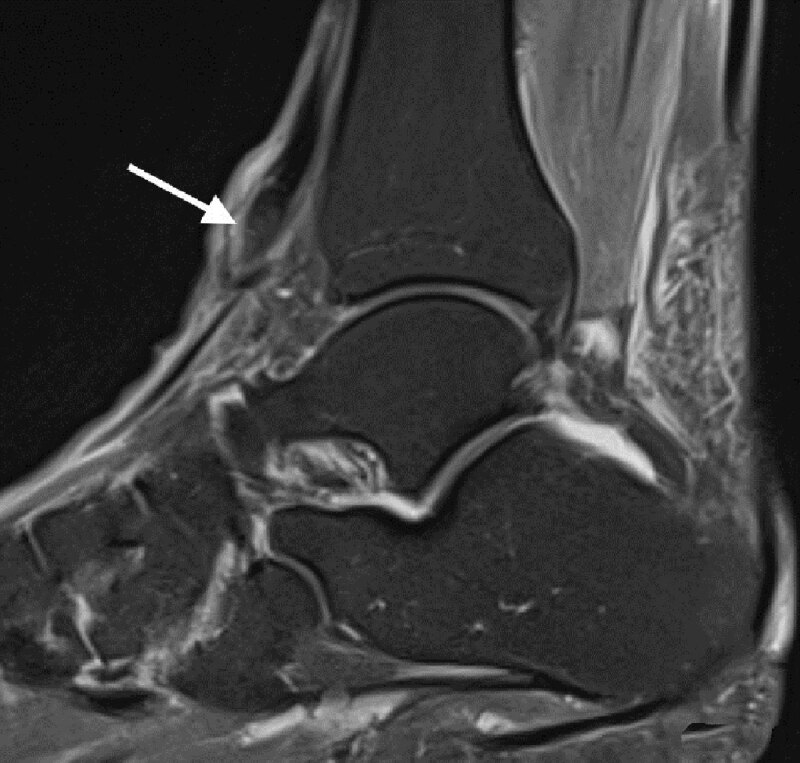


**Figure 3:**
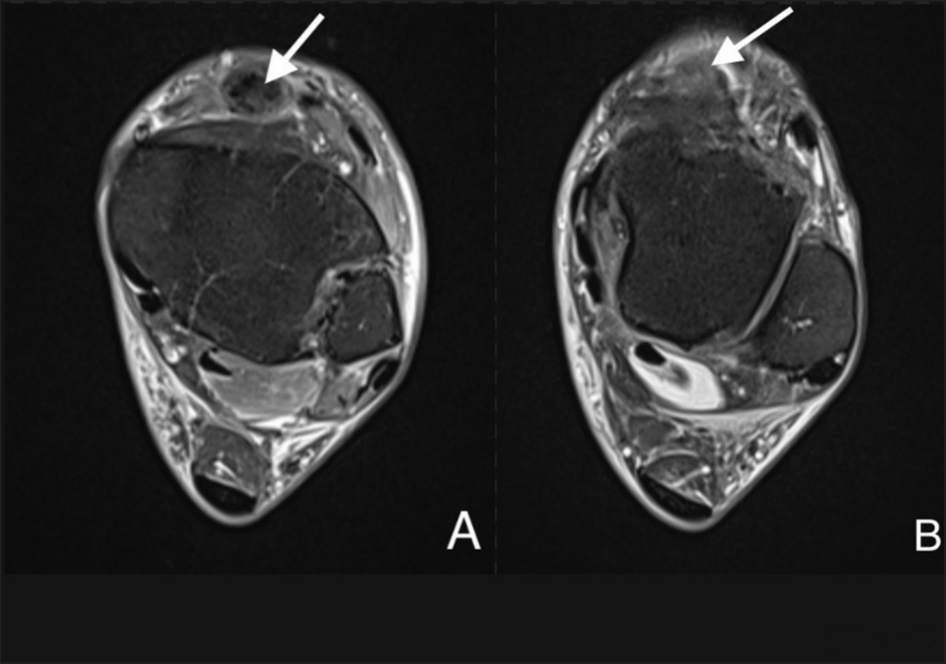


Magnetic resonance imaging (MRI) confirmed a full-thickness tear of the TAT with slight retraction of the tendon on sagittal fat-suppressed (FS) T2-weighted Image (WI) (Figure 2, arrow). Axial FS T2-WI showed a swollen heterogeneous TAT above the ankle joint (Figure 3A, arrow) and absence of the TAT below the tibiotalar joint (Figure 3B, arrow).

## Comments

TAT courses at the medial compartments of the superior and inferior retinacula. It inserts at the medial cuneiform bone and the base of the first metatarsal bone. Dorsiflexion of the foot is its primary function. Diagnosis of TAT rupture should be suspected clinically if a triad of a ‘pseudotumour’ of a ruptured tendon, loss of tendon contour, and reduced dorsiflexion is present [1].

Spontaneous rupture occurs predominantly in the elderly with pre-existing tendinopathy. The tear can go clinically unrecognized because of preserved active dorsiflexion of the foot – although significantly weakened – due to residual integrity of the digitorum longus and hallucis longus tendons.

Patients may present with a focal soft tissue swelling above the anterior aspect of the ankle joint, because of tendon retraction. Although careful comparative clinical examination may suggest the diagnosis, by revealing a tendon gap, imaging is often required to confirm the diagnosis of tendon rupture and to exclude the diagnosis of a soft tissue tumor.

Ultrasound shows focal tendon thickening and heterogeneity, with loss of the normal fibrillar pattern above the level of the tendon tear and absence of the tendon between the superior and inferior extensor retinaculum. Power doppler may show hypervascularity. Proximal tendon thickening with increased signal intensity on T2-WI, and tendon discontinuity at the level of the tibiotalar joint are the diagnostic imaging features on MRI.

Treatment is usually conservative in elderly patients with extensive comorbidities. The use of an ankle-foot orthosis, bracing, and shoe adaptations are recommended.

In conclusion, although diagnosis of TAT rupture depends on the clinical triad, elderly patients with spontaneous TAT tears may only present with a soft tissue swelling at the ankle joint, which may be misinterpreted as a soft tissue tumor. Imaging is pivotal to define the pseudotumoral nature of this lesion.
